# Fordyce Happiness Program and Happiness in Mothers of Children with a Cleft Lip and Palate

**Published:** 2016-11

**Authors:** Zeinab Hemati, Fatemeh-Sadat Mosavi Asl, Samira Abbasi, Zohre Ghazavi, Davood Kiani

**Affiliations:** 1 *Nursingand Midwifery Care Research Center, Faculty of Nursing and Midwifery, Isfahan University of Medical Sciences, Isfahan, Iran.*; 2 *Department of NICU, Shahid Beheshti Hospital, Isfahan University of Medical Sciences, Isfahan, Iran.*; 3 *Department of Psychiatric Nursing, Faculty of Nursing and Midwifery, Isfahan University of Medical Sciences, Isfahan, Iran.*; 4 *Department of Psychiatric Nursing, Hajar Hospital, Shahrekord University of Medical Sciences, Shahrekord, Iran.*

**Keywords:** Cleft lip and Palate, Children, Fordyce happiness program, Mothers

## Abstract

**Introduction:**

Facial deformities and aesthetic and functional anomalies in children may be a cause of real distress in families. Problems faced by parents in coping with a child’s anomaly can be upsetting and lead parents to exhibit over-severe behavior. The present study was conducted in order to study the effect of happiness program on the happiness of the mothers of children with a cleft lip and palate.

**Materials and Methods:**

In this semi-experimental study, 64 mothers of children with a cleft lip and palate enrolled by convenience random sampling were assigned to an intervention or control group based on a simple random sampling. Then, a program of happiness training was implemented consisting of 10 sessions of 2 hours each. A demographic questionnaire and the Oxford Happiness Questionnaire were completed prior to and 2 months after the last session of intervention. The data were analyzed using descriptive and analytical statistics, consisting of a paired t-test, independent t-test and Chi-square test using SPSS version 20.

**Results:**

The independent t-test indicated a significant difference in mean happiness score after training between the intervention and control groups (P<0.05). Moreover, the paired t-test indicated a significant difference in mean happiness score between before and after training in the intervention group, although the difference was not statistically significant for the control group (P>0.05).

**Conclusion:**

In light of the efficacy of happiness training on the promotion of happiness in the mothers of children with a cleft lip and palate, this model is recommended as a healthcare intervention to decrease stress in mothers following the birth of an infant with a cleft lip and palate.

## Introduction

Combined cleft lip and cleft lip and palate occur in one per 750 white infants, while cleft lip alone occurs in approximately one per 2,500 white infants. Overall, this anomaly affects male infants more commonly than females. A variety of factors such as maternal use of certain medications, smoking, alcohol, malnutrition, vitamin deficiency, maternal mental problems, and exposure to certain toxins or chemicals contributes to development of this deformity (1,2). The prevalence of cleft lip alone depends on several factors such as ethnicity, race, geographical conditions, and economic status. For example, cleft lip alone and/or cleft lip and palate is most prevalent among Native Americans (3.7 per 1,000 live births) and least prevalent in blacks (0.3 per 1,000 live births) (3). At present, there are no comprehensive data on the frequency of disorders associated with cleft lip and palate or their association with each other. Several epidemiological studies in Iran (except west Iran) have reported varying incidence rates (3.77–77% per 1,000 live births) for this anomaly in different regions of the country (4).

Children with this deformity face many problems during development, and require a team management approach. By offering optimal services at an appropriate time and by reducing costs, team management increases the emotional and psychological support available to families (5). Parental problems at birth and difficulties in coping with the defect are primary issues in the caretaking of an infant with a cleft lip and palate.

A study by Hasanzadeh et al. demonstrated that the mothers of children with a cleft lip and palate are more frequently subject to chronic psychiatric disorders (6). The psychological problems and pressures imposed on the patients’ families (known as subjective and objective imposed pressures) cause negative effects among families and may also undermine the effectiveness of treatment in children (7).

Pritchett et al. (2010) argued that poor psychological health and weak family function are associated with each other (8). Therefore, since the families of children with a cleft lip and palate may be under stress, which may affect reception of healthcare services and development in children, then use of psychological training is urgently needed to enhance quality of life and reduce stress in these children. Happiness training is one way to minimize depression and stress because an individual evaluation of oneself and one’s life may include cognitive dimensions such as judgment on life satisfaction and/or emotional issues including mood or emotions in response to life events (9). In this regard, research has indicated that a Fordyce cognitive-behavioral training approach was effective in reducing depression in students (10). A study of the efficacy of happiness training using the Fordyce approach on depression in elderly women indicated that training in the expression pf emotions and the development of optimism and positivist thinking led to an improved attitude toward others and therefore an increased rate of happiness (11).

In light of the inadequacy of psychological studies on the parents of children with a cleft lip and palate, as well as the high incidence of this deformity in Iran and worldwide and the sociocultural differences between Iran and other countries, this study was conducted in order to investigate whether a happiness intervention can improve happiness in the mothers of children with a cleft lip and palate. Implications of this study in terms of various research, education and treatment areas may allow steps to be taken to decrease mental and family challenges in parents of children with these deformities.

## Materials and Methods

This semi-experimental study was conducted on two groups of 32 participants each. The sample size was determined to be 32 in each group according to the findings of similar studies (d=. /7s^2^, α=0.05, β=0.2) (12). Ethical approval was provided by the Research and Technology Deputy of the Isfahan University of Medical Sciences and the letter of permission was provided by the authorities of the Cleft Lip and Palate Clinic affiliated with the Faculty of Rehabilitation of the Isfahan University of Medical Sciences. The study was conducted at the Cleft Lip and Palate Clinic affiliated with the Faculty of Rehabilitation of the Isfahan University of Medical Sciences.Mothers of children with a cleft lip and palate were enrolled by simple random sampling so that the first referring individual meeting the inclusion criteria was assigned to the intervention group and the second to the control group, continuing until the desired number of participants were included in the two groups. All mothers in the intervention and control groups had a child with a cleft lip and palate. Inclusion criteria were having Iranian nationality, being literate, be able to discuss and debate in training sessions, having full mental health and consciousness (no emergency, severe conditions throughout the study), and having a child aged 0–12 years with a cleft lip and palate (because of the psychological effects on the family due to an affected child within this age range (13). Exclusion criteria were absence from two consecutive and/or one quarter of all sessions, mental retardation, and withdrawal from the study at any time.

In this study, happiness training was considered an independent variable, happiness was a dependent variable, and child’s age and gender, mothers’ education, and occupation were underlying variables. ANCONA was used to adjust for confounders.

After explaining the aims of this research and obtaining written informed consent from the participants in the two groups, the researcher completed the pretest questionnaires. Then, the training sessions were scheduled and the classrooms of the Faculty of Rehabilitation were appointed as the setting for sessions, with the participants’ consent in the intervention group.

Consisting of eight cognitive components and six behavioral components, educational materials were offered according to the Fordyce approach. The approach for each session was as follows: First session: Introducing participants to each other, reviewing the plan for the session, reviewing relevant regulations, protocol, and training techniques to increase levels of activity; Second session: Techniques for enhancing social relationships and intimacy; Third session: Techniques for expressing emotions and developing optimism and positivist thinking; Fourth session: Techniques for decreasing expectations and increasing appreciation; Fifth session: Living in the present; Sixth session: Techniques for giving value to happiness and resolving problems and negative emotions, Seventh session: Techniques for discontinuing worries; Eighth session: Techniques for enhancing creativity; Ninth session: Techniques for planning and organizing daily activities; and Tenth session: Completing the questionnaires 2 months after the ninth session. Participants were trained by a psychologist in groups and individually through speech, brainstorming, and educational aids such as PowerPoint within 2 hour session a week session a week. In addition, the participants were given the researcher’s phone number to allow further advice and support during the 2-month follow-up, if necessary. After the follow-up, patients in both groups completed the happiness questionnaire once again. It is worth mentioning that the control group underwent no intervention. However, to comply with research ethics, after completion of the intervention and administration of the questionnaires, both groups attended four seasons, although these sessions were brief for the control group. For ethical reasons, the researcher trained the mothers in both groups in a similar way after filling out the questionnaires.

The data were gathered by a questionnaire of demographic data and *Oxford Happiness *Questionnaire, developed by Argail in 1989 and consisting of 29 four-choice items. The lowest score for the Oxford Happiness Questionnaire is zero and the highest score is 87 (10). This questionnaire has previously been used in a number of studies in Iran, with a reported Cronbach’s alpha of 93% (14,15). The data were analyzed using descriptive and analytical statistics (paired t-test [to investigate within-group significance], independent t-test [to investigate inter-group significance] and Chi-square) using SPSS 20.

## Results

The mean age of the mothers in the treatment and control groups was 33.3±6.3 and 33.5±5.8 years, respectively. The mean age of the children in the intervention and control groups was 6.34±3.37 and 5.03±3.36 years, respectively, with no significant difference between the two groups by independent t-test (P>0.05). Moreover, there was no significant difference in the mothers’ level of education and occupation, or number of children between the two groups (P>0.05) (Table.1).

An independent t-test indicated a significant difference between the mean happiness scores of the two groups after training (P=0.04), while the difference was not statistically significant in the two groups before training (P=0.23). A paired t-test exhibited a significant difference in the mean happiness score between before and after training in the intervention group (P=0.004), but the difference was not statistically significant in the control group (P=0.91) (Table .2).

**Table 1:** Demographic characteristics in the two groups

**Demographic variable **	**Intervention group**	**Control group**	**P-value**
Mother, Mean age (yr) ±SD	33.3±6.3	33.5±5.8	0.8
Child, Mean age (yr) ±SD	6.34±3.37	5.03±3.3	0.12
Child sex, N (%)			
Male	20 (62.5)	18 (56.3)	0.7
Female	12 (37.5)	14 (43.7)	
Mother education level, N (%)			0.3
Elementary	3 (9.4)	6 (18.8)	
Secondary	4 (12.5)	6 (18.8)	

**Table 2:** Comparison of mean (standard deviation) scores of happiness in the intervention and control groups before and after training

Time	Group (Mean±SD)		P-value
	Intervention	Control	Independent t-test
Before training	70.01±12.6	74.03±14.1	>0.05
After training	79.6±11.5	73.6±11.6	<0.05
p-value (paired sample t-test)	<0.05	>0.05	

Furthermore, an independent t-test indicated that the mean happiness score was not significantly associated with the children's gender in the intervention (P=0.59) and control (P=0.87) groups, the children's age in the intervention (P=0.85) and control (P=0.83) groups, or the mothers' occupation in the intervention (P=0.22) and control (P=0.12) groups. Analysis of variance indicated that the happiness score was significantly associated with the mothers' age in the control group (P=0.03) and the number of children in the intervention group (P=0.02).

## Discussion

The present study was conducted to investigate the effect of happiness training on the rate of happiness in the mothers of children with a cleft lip and palate. In this study, there was no significant difference between the intervention and control groups in terms of demographics, including children’s age and gender, mothers’ age and education level, as well as the number of children. In the present study, despite no significant difference in the mean happiness score of the controls prior to and after training, the difference was significant between before and after the intervention in the treatment group.

Facial deformities can be a cause of distress for parents, with lip deformities causing the most severe response. These families require support and encouragement from healthcare teams and advice to engage in activities that provide a normal life for the affected child (16). A study of the effect of cleft lip and palate on the family demonstrated that stress, anxiety, and psychological problems were more severe in families with more affected children (17). In addition, another study demonstrated that the mothers of infants with a cleft lip and palate experienced symptoms of post-traumatic stress more frequently than those of healthy infants (18).

A study by Nassab on the efficacy of happiness training using the Fordyce approach on depression in elderly women indicated that depression in the intervention group was significantly reduced compared with the control group. Moreover, training in the expression of emotions and the development of optimism and positivist thinking led to an improved attitude toward others, and hence happiness increased (11). In addition, a study by Narmashiri et al. investigating happiness training using the Fordyce approach in relation to difficulties with adjusting to excitement and insensible beliefs in adolescents in rehabilitation centers demonstrated that eight sessions of happiness training within a period of 8 weeks resulted in a significant decrease in difficulties associated with adjusting to excitement and resulted in the recovery of physical health, an increase in rate of happiness, an acceptable occupational and academic performance, and improvement in social interactions and interpersonal function (19). Finally, a study by Heydarabadi on group reality therapy and happiness in mothers with blind children indicated that 10 sessions of group counseling led to an increase in happiness and tirelessness in the mothers (20).

The findings obtained could be explained by the fact that mothers of children with a cleft lip and palate often ignore their most fundamental needs because they are so engaged in their children’s problems and fail to embrace the reality. Hence, attending the training sessions in the present study may contribute to improvement in the mothers’ ability to embrace reality and focus on their own primary needs, including happiness. According to the present study findings, the mean happiness score was not significantly different prior to training between the control and intervention groups, although the difference between the two groups became significant after training, a finding which is consistent with a study reported by Nikmanesh et al. The Nikmanesh study, investigating the effect of positivist thinking in decreasing depression, stress, and anxiety in adolescents in rehabilitation centers, indicated that positivist thinking training such as happiness, good mood, positive emotions, hopefulness, and satisfaction could result in feeling healthy and happy in the intervention group (21).

In the present study, appropriate training caused the mothers of children with a cleft lip and palate to recognize themselves and their children further, to embrace realities through identifying their strengths and weaknesses, and to cope with existing circumstances more appropriately, leading to a feeling of happiness. The nature of the group training approach adopted in the sessions, where individuals could find out that others may have problems similar to their own, contributed to the mothers’ embracing reality and coping with current conditions. A study by Feicht et al. of web-based happiness training and the physical and psychological parameters of personnel demonstrated that web-based, 7-week happiness training significantly increased happiness and life satisfaction compared with the control group, such that that the change persisted up to 4 weeks after the intervention (22). A study reported by Rostami et al. on the efficacy of positivist thinking on happiness in adolescents with hearing impairment indicated that attending eight sessions of positivist thinking skills training caused an increase in happiness in the intervention group compared with controls (23). In the present study, training in positivist skills and the techniques adopted in the training sessions sought to reinforce and improve positive communication and to increase happiness, and helped mothers know themselves better, recognize their positive experiences, and understand the role of such experiences in promoting respect to oneself. In addition, this approach caused an increase in the rate of happiness in the intervention group compared with the control group through highlighting talents and capabilities, optimizing joys, considering positive issues and emotions further, and preventing negative emotions from entering personal territory as well as increasing positive communications as a principle of positivism.

A study by Abolghasemi et al. of the efficacy of happiness training using the Fordyce approach on happiness and depression in women indicated that attending six sessions of Fordyce cognitive-behavioral training increased life satisfaction and happiness in the intervention group compared with controls (24).

In the present study, the emphasis on techniques for coping with negativist emotions and discontinuing concerns in the training sessions could contribute to the rate of happiness. Pearson's correlation coefficient indicated no significant association between any demographic variables and happiness in the treatment or control groups. Regarding the significant contribution of this deformity to the child’s appearance from birth, parents with any educational level, age, or occupation were found to be involved to a similar extent, because the long-term treatment in these children as well as the substantial costs imposed on the parents can affect their mental and psychological conditions under any circumstances.

The most notable limitation of the present study was a failure to schedule the training sessions according to the mothers' working hours. Because absence from more than two sessions was considered one of the exclusion criteria, some participants were very likely to be excluded. Therefore, the researcher held sessions at the weekends to try to allow most participants to attend the sessions.

## Conclusion

In light of the present study findings and the positive effect of happiness training based on the Fordyce approach, and the focus on the role of nurses in rehabilitation and that of mothers in the family, use of this form of training as an economical and accessible approach is recommended for mothers of children with a cleft lip and palate in rehabilitation centers.
